# Bayesian Modeling to Project the National and Regional Burden of Rheumatic Heart Disease in Brazil Till 2050

**DOI:** 10.5334/gh.1504

**Published:** 2025-12-10

**Authors:** Pedro Rafael Vieira de Oliveira Salerno, Antoinette Cotton, Zhou Chen, Vaibhav Shah, Gabriel Tensol Rodrigues Pereira, Sadeer Al-Kindi, Craig Sable, Antonio Luiz Pinho Ribeiro, Andrea Z. Beaton, Salil V. Deo, Bruno Ramos Nascimento

**Affiliations:** 1 Department of Medicine, NYC Health & Hospitals/Elmhurst, Icahn School of Medicine at Mount Sinai, Queens, USA; 2 Harrington Heart and Vascular Institute, University Hospitals, Cleveland, USA; 3 Case Western School of Medicine, Case Western Reserve University, Cleveland, USA; 4 Houston Methodist Hospital, Houston, USA; 5 Division of Cardiology, Children’s National Hospital, Washington DC, USA; 6 Department of Internal Medicine, Hospital das Clinicas, Federal University of Minas Gerais, Belo Horizonte, Brazil; 7 Division of Pediatric Cardiology, Cincinnati Childrens Hospital, University of Cincinnati, Cincinnati, USA; 8 Louis Stokes Cleveland VA Medical Center, Cleveland, USA; 9 School of Health and Wellbeing, University of Glasgow, Glasgow, United Kingdom; 10 Departamento de Clínica Médica, Faculdade de Medicina da Universidade Federal de Minas Gerais, Belo Horizonte, Brazil; 11 Serviço de Hemodinâmica, Hospital Madre Teresa, Belo Horizonte, Brazil

**Keywords:** rheumatic heart disease, cardiovascular epidemiology, Brazil

## Abstract

**Background:**

Rheumatic heart disease (RHD) remains a significant public health concern in middle- to low-income countries. Despite advancements in healthcare access and public health measures in Brazil, future projections of RHD burden are essential to guide policy-making. Thus, we projected the national and regional burden of RHD in Brazil through 2050.

**Methods:**

Annual prevalence counts and disability-adjusted life years (DALYs) for RHD from 2000 to 2021 were extracted from the 2021 Global Burden of Disease (GBD) dataset for 10-year age brackets (5–74 years). Age-standardized prevalence (asPR) and DALYs rates (asDALYs) per 100,000 were calculated nationally and regionally. Bayesian age-period-cohort models were used to project trends through 2050, with results reported as medians (25^th^, 75^th^ percentiles) and estimated annual percentage changes (EAPCs).

**Results:**

From 2000 to 2021, Brazil’s population grew by 27%. Nationally, the asPR declined slightly from 1,503 to 1,495 per 100,000 [EAPC: –0.04% (95% CI: –0.05, –0.03)], with reductions observed in most regions. However, increases were noted in the North [EAPC: 0.14% (95% CI: 0.13, 0.15)] and Northeast [EAPC: 0.02% (95% CI: 0.01, 0.03)]. Males experienced greater reductions [EAPC: –0.16% (95% CI: –0.19, –0.13)] compared to females, who showed a slight increase [EAPC: 0.05% (95% CI: 0.03, 0.07)]. Projections indicate that asPR will decline nationally to 1,418 per 100,000 by 2050 [EAPC: –0.20% (95% CI: –0.20, –0.19)], with the South and Central West regions reducing the most.

The asDALY rates declined from 142 to 104 per 100,000 [EAPC: –1.58% (95% CI: –1.69, –1.46)] during 2000–2021, with all regions showing decreases, particularly the Southeast [EAPC: –1.83% (95% CI: –1.98, –1.69)]. Nationally, projections suggest further reductions to 75 per 100,000 by 2050 [EAPC: –1.17% (95% CI: –1.22, –1.11)].

**Conclusion:**

The burden of RHD in Brazil has decreased nationally and regionally over recent decades. Projections suggest that these trends will continue.

## Introduction

Rheumatic heart disease (RHD) is the most prevalent acquired heart disease among children and teenagers. It results from inadequately treated streptococcal infections—markedly in the pharynx—triggering single or repeated episodes of *acute rheumatic fever* (ARF) that ultimately results in an immune-mediated process that affects heart valves (1). The disease is intrinsically derived from suboptimal access to healthcare, overcrowding, and poor sanitation, thus having a strong association with the individuals’ socioeconomic conditions (2).

Considering this, the burden of RHD is unevenly distributed across the globe and concentrated in regions such as South Asia, Eastern sub-Saharan Africa, Western sub-Saharan Africa, and some low-income Latin American areas—where there was a slight increase in age-standardized prevalence rates (asPR) since 1990—and the global reduction of mortality rates and disease burden observed in the past four decades varied widely according to socioeconomic status (3). In the macro-region of tropical Latin America, composed of Brazil and Paraguay, RHD prevalence in 2021 was 1,267 per 100,000 inhabitants, a relatively stable pattern compared to 2019, resulting in approximately 3,047,102 prevalent cases. The age-standardized disability-adjusted life years (asDALY) rates, on the other hand, showed an over 30% decrease since 1990, being 93.7 per 100,000 in Brazil in 2021, depicting a decreasing trend of morbidity and mortality (4,5).

However, even within countries, a regional disparity can be captured by estimation models, such as observed in Brazil, a country with continental dimensions and a wide variation in sociodemographic status, access, and quality metrics related to healthcare (6). Besides these known socioeconomic disparities, health crises such as the COVID-19 pandemic reinforced regional differences, in terms of number of healthcare providers, hospital beds and intensive care facilities per inhabitant; the North and Northeast regions—the regions with the worst healthcare metrics—were hardest hit in terms of mortality and other unfavorable health outcomes (7).

For this reason, and considering the impact of RHD on Brazilian health metrics—with over 50% of valve heart surgeries in the public health system resulting from its late sequelae—it is of utmost importance to investigate the predicted impact of the disease for the next decades, assessing possible regional particularities (8,9). The length and spatial extent of epidemiological data series, available for Brazil from multiple sources—including its robust administrative databases and mortality information systems—are particularly important for investigating annual and inter-annual patterns of disease. Such forecasts, regionally decomposed, may inform health systems about the ideal strategies and optimal policies to mitigate the impacts of this neglected disease of the heart. Therefore, in this study, we aimed to study the observed burden of RHD in Brazil at the regional level and use this data to project the expected RHD burden till 2050.

## Methods

Data and codes used to produce these results can be provided by the corresponding author upon reasonable request or downloaded from the authors’ GitHub account.

### RHD estimates

We obtained the RHD estimates for Brazil from the recently published 2021 Global Burden of Disease (GBD) (10). The GBD consists of a global consortium with more than 12,000 collaborators and provides harmonized information regarding morbidity and mortality, attributable to various health conditions and risk factors from 204 countries and territories worldwide. The recently updated 2021 version of GBD provided public access to data through its online Global Health Data Exchange query tool (GHDx, http://ghdx.healthdata.org/gbd-results-tool). For Brazil, and for some other countries, state-level data is provided by the GBD.

Using their data portal, we collected the annual prevalence and disability-adjusted life years (DALYs) counts attributed to RHD for each state in Brazil for the following 10-yearly age bracket intervals (5–14, 15–24, 25–34, 35–44, 45–54, 55–64, 65–74 years) between 2000 and 2021. We then combined state-level data to calculate and report the regional- and national-level data. From the available epidemiological measures and time-series metrics related to RHD, we chose these two metrics (prevalence, DALYs), as together they provide a representative picture of the overall disease burden.

### Population estimates

From the 2021 GBD tool, we obtained the mid-year population estimates for the same 10-yearly age intervals (5–14, 15–24, 25–34, 35–44, 45–54, 55–64, 65–74 years) (10). The 2021 GBD has sourced population counts from the Brazilian Institute of Geography (*Instituto Brasileiro de Geografia e Estatística* (IBGE)), which manages census information in Brazil (11). As with the prevalence and DALYs, we collected this information for each state and then combined data to obtain regional- and country-level estimates. We collected the above data for the whole population and then separately for males and females.

### Statistical analysis

We first utilized the 2000 mid-year population estimates to calculate the observed yearly asPR and asDALY rates (per 100,000 inhabitants) for RHD burden between 2000 and 2021; the 95% confidence interval (CI) for the observed asPR and asDALY rates were obtained using the delta method. We then investigated the trend over time in the observed time period by calculating the estimated annual percentage change (EAPC). The EAPC model was fitted using the generalized linear regression framework with a log-link function. For the EAPC, 95% CIs were calculated via bootstrapping with 10,000 resamples. Simply put, if the EAPC and its 95% CI are all positive, this would demonstrate a statistically significant increase in the asPR or asDALY rates over time, with negative values demonstrating a reducing trend.

We fitted Bayesian age-period-cohort models (BAPC package in the software R version 4.3.0) to the observed years (2000–2021) to estimate the projected rates for prevalence and DALYs between 2022 and 2050 (12). Age-period-cohort models allowed us to model all three components—age, period, and cohort effects—in a single regression equation (13). These models were fitted using random walk 2 priors with a log-gamma distribution. A generalized linear regression framework with a log-link and a Poisson distribution was used to model the outcome variable (in this case the asPR or asDALYs rate). For computational efficiency, we used the integrated nested Laplace approximation (INLA) method to obtain the median, 25^th^ percentile, and 75^th^ percentile values from the posterior distribution of projected asPR and asDALYs rate obtained from our model. The INLA approach is computationally quicker, does not have convergence issues, and provides results that are very similar to the Monte Carlo simulation methods. Further details regarding the model priors, methodology of obtaining the projected estimates, and model assessment are available in the supplemental section. We separately fitted these models for each region of Brazil and then aggregated the regional raw count and population data to fit these models for Brazil as a whole. For all Bayesian age-period-cohort models, we calculated the projected asPR and asDALY rates using the mid-year population in 2000 as the index for direct standardization. While age-standardized models were fitted for all regions, age bracket-specific models were also fitted for Brazil.

We used the software R version 4.3.0 (R Foundation for Statistical Computing, Vienna, Austria) for all statistical analyses. We have provided more details regarding the modeling approach and R packages used in the supplemental material. The Institutional Review Board has exempted this study as it used publicly available population-level data. As the study used population-level data, individual person consent was also not required. We conducted and reported the study according to STROBE guidelines.

## Results

### Projected population change

In 2000, Brazil had 153.9 million residents in the 5–74 years age bracket, which is expected to increase by 27% to reach 196.8 million by 2050. All regions will experience an increase in their population, with the North (78%) and South (18.4%) expected to have the largest and smallest proportional increase between 2000 and 2050, respectively. The overall population structure in Brazil is expected to age both nationally, and in each region. In Brazil, the number of residents in the 5–14 years age bracket will reduce by 30.2% while the number in the 65–74 years age bracket will increase by 312%. All regions will experience this trend of a shrinking 5–14 age bracket and an expanding 65–74 age bracket.

### Prevalence

#### Observed trends

*Overall:* In Brazil, the observed asPR rate reduced slightly from 1,503.3 per 100,000 in 2000 to 1,494.7 per 100,000 in 2021 [EAPC: –0.04% (–0.05, –0.03)]. The North [EAPC 0.14% (0.13, 0.15)] and Northeast [EAPC: 0.02% (0.01, 0.03)] regions reported a trend toward slightly increased asPR, while it reduced in the other regions (eTable 1).

*Males:* The RHD asPR rate in Brazil reduced from 1,335.4 per 100,000 in 2000 to reach 1,295.0 per 100,000 in 2021 [EAPC: –0.16% (–0.19, –0.13)] among males. All regions also reported a trend toward a reduced asPR rate between 2000 and 2021. This decline in the RHD asPR rate was numerically greatest in the South [EAPC: –0.20% (–0.23, –0.18)] and smallest in the Northeast [EAPC: –0.09% (–0.12, –0.06)] (eTable 1).

*Females:* The RHD asPR rate in Brazil increased in females from 1,672.8 per 100,000 in 2000 to reach 1,693.4 per 100,000 in 2021 [EAPC: 0.05 (0.03, 0.07)]. The South [EAPC: –0.02% (–0.04, –0.01)] was the only region in Brazil that reported a slight decline in the asPR rate among women and it tended to slowly increase in all other regions. This increase was greatest in the North [EAPC: 0.19% (0.16, 0.21)], followed by the Northeast [EAPC: 0.11% (0.09, 0.13)] (eTable 1).

#### Projected estimates

**Figure 1:**
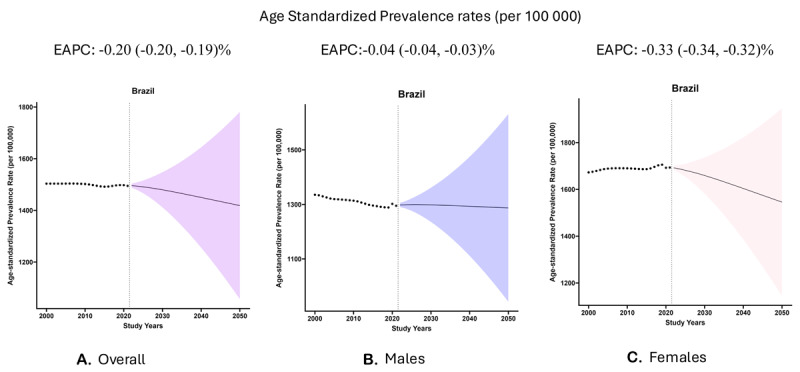
This panel of figures presents the observed (black dots) and projected (fan plot) age-standardized prevalence rate (per 100,000 residents) in Brazil. The overall rate is reported in Panel A, while Panels B and C represent the results for males and females, respectively. The line in the fan plot represents the median with the edges corresponding to the 25^th^ and 75^th^ percentile. The figure also presents the estimated annual percentage change (EAPC) (with 95% confidence interval) for the projected period (2022–2050).

*Overall:* Between 2022 and 2050, we projected that the asPR rate will reduce substantially from 1,495.5 per 100,000 to reach 1,418.5 per 100,000 [EAPC: –0.20% (–0.20, –0.19)] (Figure 1A). This reduction in asPR rates is projected to occur for all regions; the South [EAPC: –0.36% (–0.38, –0.34)] and Central West [EAPC: –0.22% (–0.23, –0.21)] are expected to have the highest reduction in rates (Table 1) (eFigure 1).

**Table 1:** The age-standardized projected prevalence rates across Brazil.


YEAR AND LOCATION	AGE-STANDARDIZED PREVALENCE RATE	EAPC	AGE-STANDARDIZED PREVALENCE RATE	EAPC	AGE-STANDARDIZED PREVALENCE RATE	EAPC
		
BRAZIL	OVERALL		MALES		FEMALES	

2022	1495.5 (1487.4, 1503.7)	–0.20 (–0.20, –0.19)	1298.0 (1290.5, 1305.6)	–0.04 (–0.04, –0.03)	1692.1 (1682.7, 1701.5)	–0.33 (–0.34, –0.32)

2050	1418.5 (1056.0, 1781.1)		1287.0 (942.6, 1631.4)		1545.7 (1144.5, 1946.8)	

**Central West**						

2022	1502.5 (1492.8, 1512.1)	–0.22 (–0.23, –0.21)	1294.9 (1285.4, 1304.3)	–0.06 (–0.07, –0.05)	1709.1 (1697.4, 1720.8)	–0.34 (–0.36, –0.33)

2050	1415.4 (1025.6, 1805.1)		1274.1 (897.9, 1650.4)		1557.2 (1113.9, 2000.5)	

**North**						

2022	1398.3 (1388.8, 1407.9)	–0.06 (–0.07, 0.05)	1252.3 (1243.2, 1261.4)	0.03 (0.03, 0.03)	1580.7 (1569.2, 1592.2)	–0.19 (–0.21, –0.18)

2050	1377.2 (985.6, 1768.9)		1262.8 (889.5, 1636.2)		1501.0 (1056.4, 1945.6)	

**Northeast**						

2022	1470.5 (1462.0, 1479.0)	–0.14 (–0.14, –0.14)	1270.8 (1262.8, 1278.9)	0.09 (0.08, 0.09)	1663.4 (1653.3, 1673.5)	–0.33 (–0.33, –0.32)

2050	1414.2 (1040.7, 1787.6)		1302.2 (940.1, 1664.4)		1519.5 (1104.2, 1934.8)	

**South**						

2022	1529.2 (1520.1, 1538.4)	–0.36 (–0.38, –0.34)	1328.0 (1319.2, 1336.8)	–0.18 (–0.19, –0.16)	1727.1 (1716.2, 1737.9)	–0.51 (–0.54, –0.48)

2050	1387.8 (1015.3, 1760.3)		1268.9 (905.5, 1632.3)		1503.8 (1093.1, 1914.6)	

**Southeast**						

2022	1521.8 (1513.1, 1530.4)	–0.20 (–0.21, –0.19)	1317.2 (1309.1, 1325.3)	–0.09 (–0.09, –0.08)	1721.5 (1711.5, 1731.5)	–0.29 (–0.30, –0.28)

2050	1442.1 (1065.7, 1818.5)		1288.7 (934.4, 1642.9)		1591.6 (1170.1, 2013.1)	


This table presents the projected median (25^th^ and 75^th^ quartile) age-standardized prevalence rate for rheumatic heart disease in 2022 and 2050 (first and last years of the projected period) for Brazil and its five constituent regions. For each group, we have also reported the estimated annual percentage change (EAPC). Confidence intervals for EAPC were calculated using bootstrap.

*Males:* In males, we projected that the asPR rate will reduce from 1,298.0 per 100,000 in 2022 to reach 1,287.0 per 100,000 in 2050 [EAPC: –0.04% (–0.04, –0.03)] (Figure 1B). Overall, most regions are projected to experience a trend toward reduced asPR rates; however, they are projected to increase slightly in the North and Northeast regions (Table 1) (eFigure 2).

*Females:* In females, we projected that the asPR rate will reduce substantially from 1,692.1 per 100,000 in 2022 to reach 1,545.7 in 2050 [EAPC: –0.33% (–0.34, –0.32)] (Figure 1C). All regions are projected to reduce their asPR rate between 2022 through 2050; the South [EAPC: –0.51% (–0.54, –0.48)] and Central West [EAPC: –0.34% (–0.36, –0.32)] are projected to have the highest reduction in their rates (Table 1) (eFigure 3).

### DALYs

#### Observed trends

*Overall:* In Brazil, the observed asDALYs rate reduced from 141.9 per 100,000 in 2000 to reach 104.2 per 100,000 in 2021 [EAPC: –1.58% (–1.69, –1.46)]. All regions reported a substantial reduction in the asDALYs rate between 2000 and 2021; the Southeast [EAPC: –1.83% (–1.98, –1.69)] and Central West [EAPC: –1.60% (–1.68, –1.51)] reported the greatest reduction while asDALYs rate reduced the least in the North region [EAPC: –1.05% (–1.15, –0.97)] (eTable 2).

*Males:* In Brazil, between 2000 and 2021, the asDALYs rate reduced from 122.6 per 100,000 to reach 88.9 per 100,000 [EAPC: –1.66% (–1.78, –1.53)]. All regions reported a reduction in their asDALYs rate; this was highest in the Southeast [EAPC: –1.91% (–2.09, –1.75)] and Central West [EAPC: –1.76% (–1.90, –1.64)] (eTable 2).

*Females:* In Brazil, between 2000 and 2021, the asDALYs rate reduced from 160.7 per 100,000 to reach 119.2 per 100,000 [EAPC: –1.52% (–1.62, –1.41)]. The asDALYs rate reduced in all regions; the Southeast [EAPC: –1.77% (–1.91, –1.63)] reported the highest reduction while the North [EAPC –0.98% (–1.07, –0.91)] reported the lowest reduction (eTable 2).

#### Projected estimates

**Figure 2:**
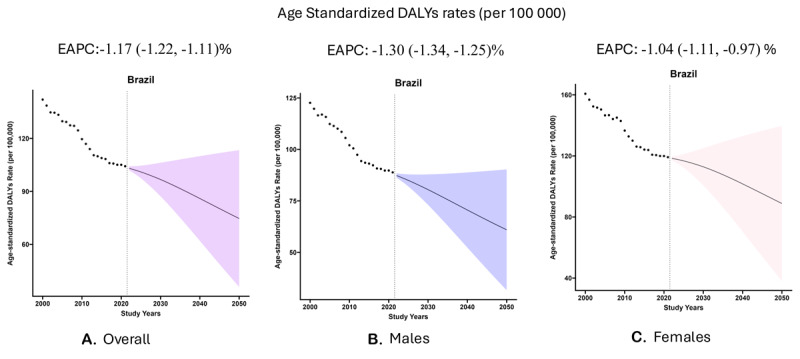
This panel of figures presents the observed (black dots) and projected (fan plot) age-standardized disability-adjusted life years rate (per 100,000 residents) in Brazil. The overall rate is reported in Panel A, while Panels B and C represent the results for males and females, respectively. The line in the fan plot represents the median with the edges corresponding to the 25^th^ and 75^th^ percentile. The figure also presents the EAPC (with 95% confidence interval) calculated for the projected period (2022–2050).

*Overall:* The RHD asDALYs rate for Brazil is projected to reduce from 103.1 per 100,000 in 2022 to reach 74.6 per 100,000 in 2050 [EAPC: –1.17% (–1.22, –1.11)] (Table 2) (Figure 2A). All regions are projected to reduce their asDALY rates between 2022 through 2050; the Northeast [EAPC: –2.18% (–2.25, –2.10)] is expected to have the highest reduction in rates (Table 2) (eFigure 4).

**Table 2:** The age-standardized projected disability-adjusted life years (DALYs) across Brazil.


YEAR AND LOCATION	AGE-STANDARDIZED DALYS RATE	EAPC	AGE-STANDARDIZED DALYS RATE	EAPC	AGE-STANDARDIZED DALYS RATE	EAPC
		
BRAZIL	OVERALL		MALES		FEMALES	

2022	103.1 (101.9, 104.2)	–1.17 (–1.22, –1.11)	87.3 (86.2, 88.4)	–1.30 (–1.34, –1.25)	118.4 (117.1, 119.8)	–1.04 (–1.11, –0.97)

2050	74.6 (35.9, 113.3)		61.0 (31.6, 90.3)		88.9 (38.2, 139.5)	

**Central West**						

2022	102.9 (101.7, 104.1)	–1.09 (–1.10, –1.08)	86.8 (85.6, 88.0)	–1.09 (–1.09, –1.08)	118.7 (117.0, 120.4)	–1.08 (–1.10–1.07)

2050	75.8 (41.0, 110.5)		63.9 (36.3, 91.5)		87.5 (40.3, 134.8)	

**North**						

2022	91.1 (90.0, 92.2)	–1.09 (–1.11, –1.07)	80.3 (79.2, 81.4)	–0.98 (–1.00, –0.96)	102.1 (100.8, 103.4)	–1.04 (–1.06, –1.03)

2050	67.3 (35.4, 99.1)		61.1 (30.6, 91.7)		76.3 (42.0, 110.6)	

**Northeast**						

2022	101.8 (100.5, 103.2)	–2.18 (–2.25, –2.10)	87.6 (86.2, 89.1)	–2.18 (–2.24, –2.12)	115.9 (114.6, 117.3)	–2.10 (–2.19, –2.00)

2050	55.5 (29.1, 81.9)		47.7 (24.6, 70.8)		64.7 (33.0, 96.4)	

**South**						

2022	103.2 (101.9, 104.5)	–0.53 (–0.57, –0.48)	87.9 (86.7, 89.1)	–0.66 (–0.69, –0.63)	118.2 (116.6, 119.9)	–0.27 (–0.31, –0.22)

2050	89.3 (36.0, 142.6)		73.3 (34.5, 112.1)		109.9 (41.0, 178.9)	

**Southeast**						

2022	106.1 (104.9, 107.4)	–0.46 (–0.51, –0.41)	88.5 (87.3, 89.7)	–0.73 (–0.76, –0.70)	123.1 (121.5, 124.7)	–0.25 (–0.31, –0.19)

2050	93.5 (37.9, 149.2)		72.4 (34.9, 109.8)		115.0 (36.8, 193.3)	


This table presents the projected median (25^th^ and 75^th^ quartile) age-standardized DALYs rate for rheumatic heart disease in 2022 and 2050 (first and last years of the projected period) for Brazil and its five constituent regions. For each group, we have also reported the estimated annual percentage change (EAPC). Confidence intervals for EAPC were calculated using bootstrap.

*Males:* In males, the asDALYs rate for Brazil is projected to reduce from 87.3 per 100,000 in 2022 to reach 61.0 per 100,000 in 2050 [EAPC: –1.30% (–1.34, –1.25)] (Table 2) (Figure 2B). All regions reported a reduction in rates, with the greatest reduction reported for the Northeast [EAPC: –2.18% (–2.24, –2.12)] (Table 2) (eFigure 5).

*Females:* In females, the asDALYs rate is projected to reduce from 118.4 per 100,000 in 2022 to reach 88.9 per 100,000 in 2050 [EAPC: –1.04% (–1.11, –0.97)] (Table 2) (Figure 2C). All regions are projected to reduce their asDALYs rate, with the highest reduction expected in the Northeast [EAPC: –2.10% (–2.19, –2.00)] (Table 2) (eFigure 6).

#### Results according to age brackets

Overall, for the whole country, while all age brackets reported a decline in their projected RHD prevalence rates (between 2022 and 2050), the rate of reduction was high for the younger age brackets (5–14 years, 15–24 years) and gradually reduced across ages (Table 3). Among males, while the younger age brackets reported a reduction in the prevalence rates over time, the prevalence rates for the 45–54, 55–64, and 65–74 years age brackets are actually projected to increase slightly between 2022 and 2050. In females, however, the projected prevalence rates for all age brackets are expected to reduce. Similarly, for Brazil, DALYs are projected to reduce for each studied age bracket between 2022 and 2050 (Table 4). Such results were also consistently observed for both sexes.

**Table 3:** Projected prevalence rates of RHD in Brazil for each age bracket.


AGE GROUP	YEAR	OVERALL	EAPC	MALE	EAPC	FEMALE	EAPC

		AGE-STANDARDIZED RATES PER 100,00 (95%CI)					

5 to 14 years	2022	748.7 (741.1, 756.3)		685.4 (678.0, 692.9)		814.8 (806.3, 823.3)	

	2050	703.6 (524.4, 943.9)	–0.22 (–0.22, –0.22)	676.1 (498.7, 916.7)	–0.05 (–0.05, –0.05)	733.6 (544.2, 988.8)	–0.37 (–0.37, –0.37)

15 to 24 years	2022	1842.5 (1827.1, 1858.0)		1630.1 (1615.6, 1644.7)		2062.6 (2045.1, 2080.3)	

	2050	1729.3 (1324.3, 2257.8)	–0.22 (–0.22, –0.22)	1604.4 (1216.3, 2116.0)	–0.05 (–0.05, –0.05)	1856.8 (1415.6, 2434.8)	–0.37 (–0.37, –0.37)

25 to 34 years	2022	2178.9 (2161.0, 2197.0)		1879.4 (1862.9, 1896.0)		2477.6 (2456.8, 2498.6)	

	2050	2050.0 (1602.5, 2621.3)	–0.23 (–0.22, –0.23)	1849.7 (1431.9, 2388.5)	–0.06 (–0.05, –0.06)	2243.4 (1746.4, 2880.4)	0.37 (–0.37, –0.38)

35 to 44 years	2022	1895.5 (1880.0, 1911.1)		1615.2 (1601.2, 1629.4)		2166.7 (2148.7, 2184.9)	

	2050	1812.5 (1437.0, 2285.0)	–0.19 (–0.17, –0.21)	1611.1 (1265.1, 2050.6)	–0.04 (–0.06, –0.02)	2001.7 (1580.6, 2533.3)	–0.32 (–0.35, –0.28)

45 to 54 years	2022	1353.3 (1342.2, 1364.4)		1133.3 (1123.4, 1143.2)		1561.1 (1548.2, 1574.2)	

	2050	1311.7 (1048.2, 1640.3)	–0.12 (–0.16, 0.09)	1144.1 (905.7, 1444.4)	0.02 (–0.01, 0.04)	1463.6 (1165.1, 1837.2)	–0.24 (–0.27, –0.2)

55 to 64 years	2022	881.4 (874.1, 888.6)		716.0 (709.7, 722.3)		1030.3 (1021.7, 1039.0)	

	2050	863.5 (692.2, 1076.4)	–0.06 (–0.09, –0.03)	729.3 (579.2, 917.7)	0.08 (0.05, 0.11)	978.2 (781.2, 1223.9)	–0.18 (–0.19, –0.13)

65 to 74 years	2022	580.7 (576.0, 585.5)		460.9 (456.9, 465.0)		680.7 (675.0, 686.5)	

	2050	573.9 (460.2, 715.2)	0 (–0.03, 0.02)	473.1 (375.9, 595.2)	0.13 (0.11, 0.15)	653.4 (521.9, 817.2)	–0.11 (–0.13, –0.09)


This table presents the projected median (25^th^ and 75^th^ quartile) prevalence rates for each studied age bracket for rheumatic heart disease in 2022 and 2050 (first and last years of the projected period) for Brazil. For each group, we have reported the estimated annual percentage change (EAPC). Confidence intervals for EAPC were calculated using bootstrap.

**Table 4:** The projected disability-adjusted life years (DALYs) in Brazil for each age bracket.


AGE GROUP	YEAR	OVERALL	EAPC	MALE	EAPC	FEMALE	EAPC

		AGE-STANDARDIZED RATES PER 100,00 (95%CI)					

5 to 14 years	2022	43.7 (42.8, 44.6)		40.4 (39.4, 41.4)		47.3 (46.3, 48.3)	

	2050	29.0 (16.9, 49.7)	–1.45 (–1.45, –1.45)	26.6 (15.8, 44.7)	–1.48 (–1.48, –1.48)	31.8 (17.7, 57.0)	–1.41 (–1.41, –1.41)

15 to 24 years	2022	104.4 (102.6, 106.2)		95.7 (93.7, 97.8)		113.2 (111.3, 115.2)	

	2050	69.5 (41.7, 115.6)	–1.45 (–1.46, –1.45)	63.1 (38.9, 102.3)	–1.48 (–1.48, –1.48)	76.4 (43.7, 133.2)	–1.41 (–1.41, –1.4)

25 to 34 years	2022	127.3 (125.1, 129.4)		110.0 (107.7, 112.3)		144.4 (141.9, 146.8)	

	2050	88.4 (52.4, 144.4)	–1.38 (–1.43, 1.31)	74.5 (47.3, 117.3)	–1.43 (–1.46, –1.39)	102.9 (60.1, 175.9)	–1.31 (–1.37, –1.23)

35 to 44 years	2022	131.8 (129.6, 134.1)		107.0 (104.8, 109.3)		155.7 (153.1, 158.3)	

	2050	97.3 (60.5, 156.3)	–1.16 (–1.27, –1.04)	76.1 (49.2, 117.4)	–1.28 (–1.36, –1.2)	119.1 (70.6, 200.8)	–1.04 (–1.18, –0.9)

45 to 54 years	2022	124.2 (122.1, 126.3)		95.2 (93.2, 97.2)		150.8 (148.3, 153.4)	

	2050	97.0 (61.0, 153.5)	–0.87 (–1, –0.74)	70.8 (46.3, 108.0)	–1.06 (–1.16, –0.96)	123.7 (73.9, 207.0)	–0.69 (–0.85, –0.54)

55 to 64 years	2022	102.3 (100.1, 104.5)		96.3 (94.3, 98.3)		147.2 (144.7, 149.7)	

	2050	102.5 (64.5, 162.7)	–0.58 (–0.69, –0.47)	75.8 (49.8, 115.0)	–0.82 (–0.92, –0.72)	127.9 (76.6, 213.2)	–0.38 (–0.5, –0.26)

65 to 74 years	2022	123.2 (121.1, 125.3)		102.7 (100.5, 104.8)		139.8 (137.5, 142.2)	

	2050	106.9 (67.3, 169.7)	–0.39 (–0.45, –0.34)	84.6 (55.7, 128.3)	–0.61 (–0.68, –0.55)	126.6 (75.9, 211.1)	–0.21 (–0.27, –0.16)


This table presents the projected median (25^th^ and 75^th^ quartile) DALYs rates for each studied age bracket for rheumatic heart disease in 2022 and 2050 (first and last years of the projected period) for Brazil. For each group, we have reported the estimated annual percentage change (EAPC). Confidence intervals for EAPC were calculated using bootstrap.

## Discussion

Combining observed and modeled data from the GBD study and the population census, our study fitted into Bayesian age-period-cohort models and showed that the burden of RHD—an important and preventable cause of mortality and morbidity in Brazil—has significantly reduced nationally over the past decades, albeit with a differential regional pattern, with mildly increasing asPR in the North and Northeast regions, reflecting the socioeconomic determinants of the disease. The results were consistent for prevalence and DALY metrics. Using observed data to inform our mathematical model, this reduction trend is projected to continue, with further declines in asPR and asDALY rates until 2050. Steeper asPR EAPCs are expected in the South, Southeast, and Central-West regions, while the North and Northeast regions are also projected to experience reductions, albeit at a slower pace. Age-stardardized DALY rates, however, are projected to have a greater reduction in the Northeast.

In the past decades, there have been several improvements in RHD care in Brazil, not only with actions and policies directly targeting the disease but also with overall improvements related to socioeconomic structure and universal access to healthcare, especially at the primary care level (14,1). There have been great improvements in cardiovascular care since the early 1990s, with the creation and further development of the Unified Health System (*Sistema Único de Saúde* (SUS), in Brazilian Portuguese), following the issue of the 1988 Constitution, which turned health into a universal right and an undeniable responsibility of the government in its three levels: city, state and federal (15). Comprising a complex network of primary, secondary, and tertiary healthcare systems, with actions and policies co-funded by these different administrative levels, the SUS is currently the exclusive health coverage for over 71% of the Brazilian population (15). Beyond improving access to healthcare, the SUS has structured comprehensive care systems, developed targeted policies and strategies, and established extensive reach through the Family Health Program. This national primary care policy aims to replace parts of the traditional specialist-driven model with a community-based approach, fostering strong connections between the healthcare system and the population it serves (16). As examples of its effectiveness, in over 30 years, infant mortality has dropped by 65%, hospital admissions associated with diabetes or stroke decreased by a quarter, and the proportion of underweight children < 5 years old has fallen by almost 70%. Thus, there is a clear parallel impact on the overall determinants of RHD, as well as on disease management (16).

Improvements toward universal access and a community approach have led to several strategies aimed at improving control of common risk factors and cardiovascular disease prevention (15). Furthermore, the community approach favored control strategies with a striking impact on RHD burden, especially those focused on health promotion—one of the pillars of the Brazilian SUS (16). Trends depicted by our study, such as the observed and projected reduction of RHD prevalence and DALY, especially in younger ages, partially result from better access to pediatric care, leading to earlier detection and treatment of pharyngitis (including the wide availability of antibiotics), stricter clinical follow-up, and inclusion in primary and secondary prophylaxis programs (17,18). These family-centered approaches are available in primary care, facilitating their implementation and favoring adherence over time. Additionally, RHD metrics are directly impacted by the socioeconomic improvement observed in Brazil in the past three decades, positively affecting determinants beyond access to healthcare such as overcrowding, housing, and poor sanitation (19,20).

Socioeconomic development, however, has always been unevenly distributed in Brazilian territory. Given the patterns of colonization, industrial, commercial, and agricultural development, the Northeast and North regions are historically in the lowest bounds of sociodemographic markers. In this sense, availability and quality of healthcare are also impacted because of such discrepancies. The numbers of hospital beds (2.14 and 2.53 per 1,000 inhabitants), intensive care beds (0.9 and 1.5 per 10,000 inhabitants), and physicians (1.45 and 1.93 per 10,000 inhabitants) in the North and Northeast are in the fifth and fourth positions among the five Brazilian regions, respectively (6). Thus, during stressful health events such as the COVID-19 pandemic, these discrepancies were accentuated, and Northern and Northeastern states with worse sociodemographic indexes and less structured health infrastructure were hardest hit by the sanitary crisis, resulting in higher infectivity, overall and cardiovascular mortality, and out-of-hospital deaths (7,21). Not surprisingly, these regions had a still increasing RHD prevalence in the past decades, and the projected estimates suggest a slower pace of reduction until 2050, although the DALY rates are projected to have an universally steep downward trend, possibly mainly driven by the reduction in premature mortality. These results reinforce the need for targeted actions and policies for these areas in the upcoming decades to mitigate the RHD burden, in addition to other cardiovascular conditions.

In parallel with the universally decreasing prevalence and DALY rates denoted by our data, the reduction estimates are more pronounced in younger ages—probably resulting from a mix of socioeconomic improvement, primordial and primary prevention—compared to older ages, meaning more adults will be living with late RHD sequelae in the upcoming decades. Considering this, the universality of the Brazilian Unified Health System provides access to advanced cardiovascular care including percutaneous procedures and valve surgery (15). Despite the challenges related to waiting queues and uneven regional provision of resources, the Brazilian situation in this setting is better than most of sub-Saharan Africa and several Latin American and Asian endemic regions. Considering the current (> 50% of valve surgeries in the SUS) and the expected burden of advanced rheumatic valve disease, it is of utmost importance to reinforce investments in tertiary care and recovery (9,8). The Brazilian Cardiovascular Statistics, compiling data from national databases, reported a near –30% drop in public expenses with hospitalizations for valve heart surgeries from 2019 to 2021 (from $42,835,735 to $30,108,266 per year), and a stable pattern for mitral balloon commissurotomy (from $447,960 to 456,066,06), despite the increasing demand (8). Furthermore, the reimbursement for some advanced cardiac procedures does not cover hospital expenses, limiting their availability to teaching and university hospitals, such as the case of mitral commissurotomy. Additionally, there was a shortage of cardiac valves for the public system in the past years—derived from a gap between the cost and the reimbursement of the devices—which was partially overcome, but limited the wide utilization of high-end bioprostheses (8). As there are more number of patients living with advanced RHD, efforts should be made at different government levels, supported by the emerging epidemiological/clinical data and registries under development, to boost investments in secondary and tertiary care, increase the availability of resources for less developed areas, as well as to reinforce and empower the existing programs at the primary level. These should include actions focused on older ages, linked to adherence to clinical treatment, post-surgical and recovery counseling.

Although there is no national health policy specifically targeting RHD in Brazil, there is a growing number of programs, frequently linked to universities and research initiatives, aimed at education and awareness, early detection, risk stratification, and treatment of RHD (22,23,24,25). Local screening programs in schools and primary care, which emerged in 2014 in the Southeastern state of Minas Gerais, found for the first time a high prevalence of latent RHD in Brazil, with a considerably higher burden among females, increasing with age, as well as high rates of undiagnosed advanced disease (26,27). These data created opportunities for different customized interventions, such as educational programs for patients and families, training modules for healthcare providers aimed at the dissemination of knowledge, and referral programs for patients at highest risk for progression. As also reinforced by this analysis, the higher prevalence and DALY rates among females and the slower projected burden reduction point toward the need for sex-specific interventions. Some of these customized approaches have also been tested, such as education for pregnant and postpartum women with RHD, and echocardiographic screening during late pregnancy in underserved neighborhoods (28). Their effectiveness in terms of clinical and gestational outcomes, however, deserves further investigation.

Brazil has suffered from global issues related to RHD, such as the significant shortage of penicillin in the late 2010s, which impacted not only secondary prophylaxis but also the treatment of diseases such as Syphilis, resulting in growing prevalence and sequelae (29). Negotiation led by the Ministry of Health and local stakeholders, along with international campaigns to stimulate production and commercialization of the drug, especially for low-resourced areas, resulted in the normalization of supplies in the Brazilian territory. There is, however, a need for constant monitoring of basic supplies, as well as for the development of partnerships between universities and health authorities for a joint development of RHD programs and the translation of strategies tested in research into public policies to ultimately improve care. The level of detail that our data brings about the reduction of the RHD burden in Brazil nationally and regionally over the past decades, with a further reduction predicted for 2050, can be applied to the planning of future actions at different levels. While the uneven predicted regional pattern over time reinforces the need for customized interventions for the different regions based on health infrastructure and sociodemographic variables, the higher prevalence and DALY rates, with heterogeneous downward trends among women and individuals at older ages highlights the importance of strengthening the health strategies customized for sex- and age-specific groups. On one side, the maintenance and improvement of primary care and community-based interventions for health promotion, prevention, early detection, and prophylaxis are essential and, on the other, investments for a real universalization of tertiary care with unrestricted and customized access to advanced therapies and recovery are mandatory for future policy-making and financial planning.

### Limitations

Our study has several limitations—related both to the observed data and to the predicted estimates—that deserve to be mentioned for the interpretation of data. Firstly, we utilized data from the GBD to report our observed and projected prevalence and DALY estimates, and limitations related to its estimation methodology have been previously detailed and are primarily related to the heterogeneity of the original data sources, especially in the poorer regions of Brazil, where granular prevalence and morbidity data may be lacking (30,31). Secondly, as RHD is still a non-communicable disease, registries are not mandatory and hence it is possible that data was not captured uniformly in all regions in Brazil. Thirdly, data utilized to produce RHD estimates (administrative databases, mortality information systems, surveillance data, and qualified published studies) are prone to inadequacies related to: 1) the extent of disease misclassification and undernotification, 2) accuracy of prevalence rates among adults in low- and middle-income countries, 3) suboptimal data about rates of non-fatal outcomes and excess mortality in longitudinal studies of individuals with RHD, and 4) availability and reliability of data about latent RHD, recently incorporated in the estimation process. Finally, our Bayesian forecasting model applies mathematical approaches to observed data; therefore, future changes in factors such as socioeconomic conditions, access to healthcare, and tertiary care may alter the true burden of RHD in the decades to come. Hence, our projections should be considered along with their uncertainty and confidence intervals presented. Despite such limitations, the GBD remains one of the most reliable sources of epidemiological information, especially in low- and middle-income countries. Each iteration of the GBD data is more accurate than the prior one, especially as it pertains to RHD. However, despite the above limitations, our study is, to the best of our knowledge, the first initiative to produce robust long-term predictions about RHD burden in a Latin American country. We hope that these results may inform governments, policymakers and stakeholders about priorities to fight RHD in Brazil and reduce its large impact on public health.

## Conclusion

Between 2000 and 2021, the age-standardized burden of rheumatic heart disease has reduced in Brazil and all its regions. While the prevalence among females is substantially higher than males, both sexes have reported a gradual decline in the age-standardized prevalence rates during this period. Based on these observed trends, we forecasted that this positive trend would continue till 2050 with all regions and the nation reporting a gradual fall in the age-standardized RHD prevalence. However, the differential improvement observed across regions highlights opportunities for further improvements in public policy and healthcare delivery.

## Data Accessibility Statement

The datasets used and/or analyzed during the current study are available from the corresponding author on reasonable request. Data and codes used for results can be requested from Dr Salil V Deo or downloaded from the authors’ GitHub account.

## Additional File

The additional file for this article can be found as follows:

10.5334/gh.1504.s1 Supplementary File. Additional details of the methodology and results.

## Figures and Tables

**Table:** 


asPR	Age-standardized prevalence rate

asDALY	Age-standardized disability-adjusted life years

EAPC	Estimated annual percentage change

GBD	Global burden of disease

RHD	Rheumatic heart disease

