# Supplementary material for: Bayesian Modeling to Project the National and Regional Burden of Rheumatic Heart Disease in Brazil Till 2050
Source: Glob Heart. 2025 Dec 10;20(1):110. doi: 10.5334/gh.1504 (PMC12700146; doi:10.5334/gh.1504)
Supplement: Supplementary File. — Additional details of the methodology and results. [file gh-20-1-1504-s1.pdf]

Bayesian age-period-cohort models:

Age-Period-Cohort models are recommended by demographers as the correct method to evaluate temporal changes in data & project future rates. They suffer from an identifiability problem as there is an exact linear relationship between age, period, and cohort as one can be directly calculated from the other two. Due to this linear dependence, it is impossible to identify separate contributions of the age, period and cohort effects. The observed event rate for the data can be presented as:

$$log(\lambda_{ij}) = \alpha(age - i) + \beta(period - j) + \gamma(cohort) + \mu\ (intercept\ term)$$

While the identifiability problem can make it challenging to identify individual contributions of age, period and cohort, accurately projecting future rates is more easily possible. However, in this study, our primary aim was to be able to reliably project future event rates, which can be reliably identified using these models. The Bayesian age-period-cohort models also model non-linear changes in the observed data. However, unlike, frequentist approaches, where spline terms need to be fitted with predetermined knots or polynomial terms need to be chosen a-priori, this model does not need such inputs from the user. Hence, it chooses an equation that is best suited to the data.

Model priors: We used smoothing priors for the age, period and cohort effects. The standard choice is to use the second-order random walk (RW2) which assumes independent mean-zero normal distributions (with unknown variance) on the second differences of all time effects. The RW2 smoothing prior can be presented as: $\Delta^2\theta_i = \theta_i - 2\theta_{i-1} + \theta_{i-2} \sim N(0, \sigma^2)$ where θ represents the parameter for the i-th level. The benefits of using the RW2 prior are: 1. It helps smooth the estimates effects across adjacent periods and reduces the noise and improves interpretability. 2. It breaks the perfect collinearity between the three effects and therefore all three effects can be simultaneously introduced into the model.

Model convergence and diagnostic accuracy: We used the Integrated Network Laplace Approximation (INLA) method to approximate the posterior marginal distributions directly from the model without Markov Chain Monte Carlo (MCMC) sampling method. The INLA approach is faster, does not have any convergence concerns, and provides very similar results to the traditional MCMC approach. The model's predictive accuracy was checked by calculating the Brier score, root mean square error and mean absolute error from the observed and model predicted values between 1990 - 2021. Additionally, we graphed and compared the observed and predicted estimates between 1990 -2021 for concordance.

We refer readers to the following manuscripts for further information regarding the BAPC package and fitting BAPC models using INLA in R:

1. https://rdrr.io/rforge/BAPC/ - this webpage provides information regarding the functions present in the BAPC package.

2. Riebler A, Held L. Projecting the future burden of cancer: Bayesian age-period-cohort analysis with integrated nested Laplace approximations. Biom J. 2017 May;59(3):531-549. doi: 10.1002/bimj.201500263. Epub 2017 Jan 31. PMID: 28139001. – This is their manuscript wherein they present the details of their modeling process.

3. Supplemental material to the above paper provides R code used for the examples presented in their manuscript.

4. https://cran.r-project.org/web/packages/scoringRules/index.html - We used the scoringRules R package to calculate the model metrics

eTable 1. Model metrics for age standardized prevalence rates

	Region	MAE	RMSE	Brier
Overall	Brazil	0.01156568	0.01513212	0.0002289812
	Central-West	0.09541494	0.1105689	0.01222548
	North	0.0744963	0.09736827	0.009480581
	Northeast	0.04619837	0.05860038	0.003434004
	South	0.06535846	0.08330449	0.006939638
	Southeast	0.0303812	0.04018056	0.001614477
Male	Brazil	0.04167614	0.06008295	0.003609961
	Central-West	0.322699	0.464225	0.2155049
	North	0.2957057	0.4485231	0.201173
	Northeast	0.1216612	0.1943499	0.0377719
	South	0.1747422	0.2300906	0.05294166
	Southeast	0.09668022	0.1369257	0.01874864
Female	Brazil	0.039074	0.052678	0.002775
	Central-West	0.264882	0.405376	0.164330
	North	0.271394	0.367046	0.134722
	Northeast	0.133784	0.193661	0.037505
	South	0.169713	0.207048	0.042869
	Southeast	0.089301	0.119033	0.014169

This table presents the Mean Absolute Error (MAE), Root Mean Square Error (RMSE), and Brier score for age-standardized prevalence rates across Brazil and its regions. The MAE, RMSE and Brier score were obtained by comparing the predicted and observed age standardized prevalence rates (per 100 000) between 2000 – 2021.

eTable 2. Model metrics for the age standardized DALYs rates.

	Region	MAE	RMSE	Brier
Overall	Brazil	0.03864433	0.04721099	0.002228878
	Central-West	0.3845755	0.4617977	0.2132571
	North	0.2648715	0.3521049	0.1239778
	Northeast	0.07431098	0.1018027	0.0103638
	South	0.2671901	0.322767	0.1041786
	Southeast	0.08461705	0.1034845	0.01070905
Male	Brazil	0.04935346	0.06201713	0.003846124
	Central-West	0.4342005	0.5300069	0.2809073
	North	0.3528588	0.429196	0.1842092
	Northeast	0.1174061	0.1467734	0.02154243
	South	0.3959647	0.4798344	0.2302411
	Southeast	0.1126377	0.1390036	0.019322
Female	Brazil	0.08115052	0.09564756	0.009148456
	Central-West	0.5865913	0.7078791	0.5010929
	North	0.4271657	0.5523002	0.3050355
	Northeast	0.1901208	0.2402597	0.05772471
	South	0.4018628	0.4779986	0.2284827
	Southeast	0.1568849	0.1924731	0.03704589

This table presents the Mean Absolute Error (MAE), Root Mean Square Error (RMSE), and Brier score for age-standardized disability adjusted life years (DALYs) rates across Brazil and its regions. The MAE, RMSE and Brier score were obtained by comparing the predicted and observed age standardized prevalence rates (per 100 000) between 2000 – 2021.

eTable3. Trend of observed age standardized prevalence rate (per 100 000) in Brazil and its regions.

Year & Location	Age-standardized prevalence rate (per 100,000)	95% confidence interval (per 100,000)	Age-standardized prevalence rate (per 100,000)	95% confidence interval (per 100,000)	Age-standardized prevalence rate (per 100,000)	95% confidence interval (per 100,000)
Brazil	Overall		Males		Females	
2000	1503.3	1501.3, 1505.2	1335.4	1332.8, 1338.0	1672.8	1669.9, 1675.7
2021	1494.7	1492.9, 1496.5	1295.0	1292.6, 1297.4	1693.4	1690.7, 1696.0
EAPC (95% CI)	-0.04 (-0.05, -0.03)		-0.16(-0.19, -0.13)		0.05(0.03, 0.07)	
Central West						
2000	1515.6	1508.2, 1523.0	1334.7	1324.9, 1344.6	1695.3	1684.3, 1706.4
2021	1500.8	1494.3, 1507.3	1291.1	1282.6, 1299.6	1710.5	1700.8, 1720.4
EAPC (95% CI)	-0.05(-0.06, -0.04)		-0.17(-0.19, -0.13)		0.05(0.03, 0.07)	
North						
2000	1354.5	1347.8, 1361.3	1284.6	1275.4, 1293.9	1514.8	1504.6, 1525.0
2021	1394.5	1388.8, 1400.2	1247.3	1239.7, 1254.9	1579.2	1570.6, 1587.8
EAPC (95% CI)	0.14(0.13, 0.15)		-0.14(-0.17, -0.09)		0.19(0.16, 0.21)	
Northeast						
2000	1466.2	1462.5, 1469.8	1291.6	1286.8, 1296.5	1634.1	1628.7, 1639.5
2021	1472.8	1469.5, 1476.1	1269.2	1264.8, 1273.7	1668.8	1663.8, 1673.7
EAPC (95% CI)	0.02(0.01, 0.03)		-0.09(-0.12, -0.06)		0.11(0.09, 0.13)	
South						
2000	1556.6	1551.5, 1561.7	1379.3	1372.5, 1386.2	1730.6	1723.0, 1738.2
2021	1527.4	1522.5, 1532.2	1325.7	1319.2, 1332.1	1726.3	1719.0, 1733.5
EAPC (95% CI)	-0.10(-0.12, -0.09)		-0.20(-0.23, -0.18)		-0.02(-0.04, -0.01)	
Southeast						
2000	1532.7	1529.7, 1535.7	1357.7	1353.7, 1361.8	1701.0	1696.6, 1705.4
2021	1520.5	1517.7, 1523.3	1314.3	1310.6, 1318.1	1721.5	1717.4, 1725.8
EAPC (95% CI)	-0.06(-0.07, -0.04)		-0.18(-0.21, -0.15)		0.04(0.02, 0.07)	

This table presents the age standardized prevalence rate (per 100 000) during the observed years of the data (2000-2021). Results are presented as overall and separately for males and females. It also reports the estimated annual percentage change (with 95% confidence interval) calculated for this period.

eTable 4. Trend of observed age standardized disability adjusted life-years rate (per 100 000) in Brazil and its regions.

Year & Location	Age-standardized DALYs rate (per 100,000)	95% confidence interval (per 100,000)	Age-standardized DALYs rate (per 100,000)	95% confidence interval (per 100,000)	Age-standardized DALYs rate (per 100,000)	95% confidence interval (per 100,000)
	Overall		Males		Females	
Brazil						
2000	141.9	141.4, 142.5	122.6	121.9, 123.4	160.7	159.8, 161.6
2021	104.2	103.8, 104.7	88.9	88.3, 89.5	119.2	118.5, 119.9
EAPC (95% CI)	-1.58 (-1.69, -1.46)		-1.66 (-1.78, -1.53)		-1.52 (-1.62, -1.41)	
Central-West						
2000	144.3	142.1, 146.6	123.1	120.1, 126.1	165.4	162.0, 168.9
2021	105.3	103.7, 107.0	89.2	87.0, 91.4	121.1	118.6, 123.6
EAPC (95% CI)	-1.60 (-1.68, -1.51)		-1.76 (-1.90, -1.64)		-1.48 (-1.56, -1.40)	
North						
2000	111.3	109.4, 113.3	98.8	96, 101	124.0	121.1, 126.9
2021	92.7	91.3, 94.2	81.7	79.8, 83.6	103.7	101.6, 105.9
EAPC (95% CI)	-1.05 (-1.15, -0.97)		-1.16 (-1.30, -1.04)		-0.98 (-1.07, -0.91)	
Northeast						
2000	133.2	132.1, 134.3	117.0	115.5, 118.4	148.8	147.2, 150.4
2021	104.4	103.6, 105.3	90.5	89.4, 91.7	117.8	116.5, 119.0
EAPC (95% CI)	-1.32 (-1.46, -1.21)		-1.40 (-1.56, -1.28)		-1.26 (-1.39, -1.16)	
South						
2000	139.7	137.2, 140.3	119.0	117.0, 121.1	158.1	155.8, 160.4
2021	103.3	102.1, 104.6	88.6	87.0, 90.2	117.8	116.0, 119.7
EAPC (95% CI)	-1.51 (-1.66, -1.39)		-1.57 (-1.73, -1.45)		-1.47 (-1.61, -1.34)	
Southeast						
2000	153.7	152.7, 154.6	131.8	130.5, 133.0	174.7	173.3, 176.1
2021	106.1	105.4, 106.8	88.9	88.0, 99.9	122.7	121.7, 123.8
EAPC (95% CI)	-1.83 (-1.98, -1.69)		-1.91 (-2.09, -1.75)		-1.77 (-1.91, -1.63)	

This table presents the age standardized disability adjusted life-years rate (per 100 000) during the observed years of the data (2000-2021). Results are presented as overall and separately for males and females. It also reports the estimated annual percentage change (with 95% confidence interval) calculated for this period.

eFigure1. Observed and projected age-standardized prevalence rate (per 100 000) for each region.

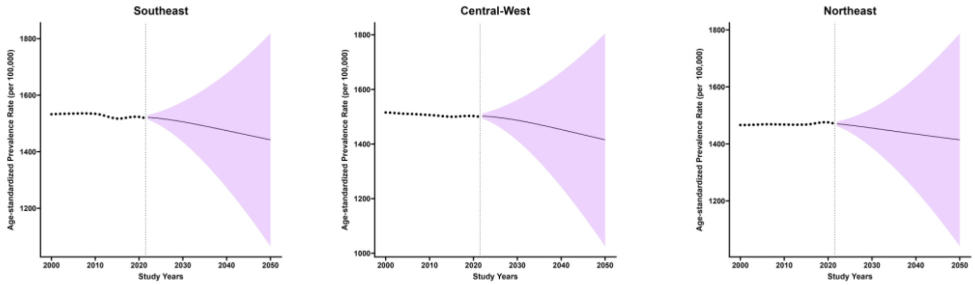

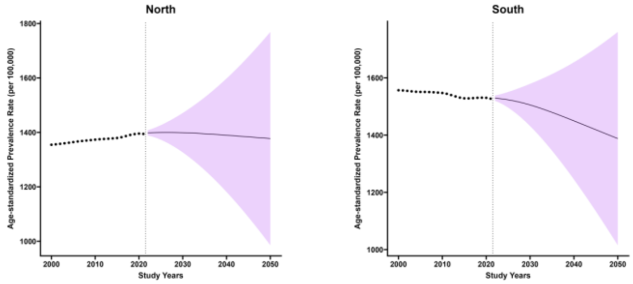

Region	Observed Period (2000-2021) EAPC (95% CI)	Projected period (2022-2050) EAPC (95% CI)
Brazil	-0.04 (-0.21, 0.13)	-0.20(-0.20, -0.19)
Central West	-0.05 (-0.22, 0.12)	-0.22(-0.23, -0.21)
North	0.14 (-0.04, 0.32)	-0.06(-0.07, 0.05)
Northeast	0.02 (-0.15, 0.20)	-0.14(-0.14, -0.14)
South	-0.10 (-0.27, 0.06)	-0.36(-0.38, -0.34)
Southeast	-0.06 (-0.22, 0.11)	-0.20(-0.21, -0.19)

This panel of figures presents the observed (black dots) and projected (fan plot) age standardized prevalence rate (per 100 000 residents) for each region in Brazil. The dots present the observed values between 2000-2021 and the fan plot presents the projected estimates between 2022- 2050.The line in the fan plot represents the median with the edges corresponding to the upper and lower bounds of the interquartile range. The table presents the estimated annual percentage change (with 95% confidence interval) calculated for each period. The figure for the Brazil is presented in Figure 1A in the main manuscript.

eFigure2. Observed and projected age-standardized prevalence rate (per 100 000) for each region in males.

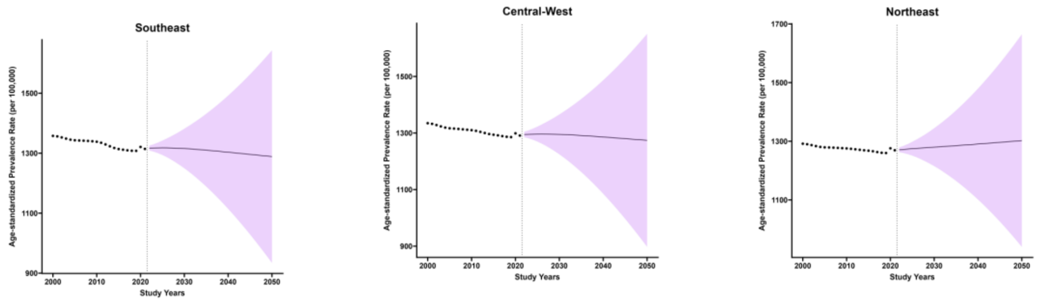

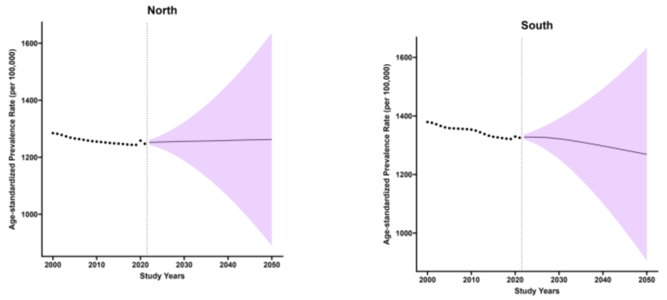

	Observed Period (2000-2021)	Projected period (2022-2050)
Region	EAPC (95% CI)	EAPC (95% CI)
Brazil	-0.16(-0.35, 0.02)	-0.04(-0.04, -0.03)
Central West	-0.17(-0.35, 0.01)	-0.06(-0.07, -0.05)
North	-0.14(-0.32, 0.05)	0.03(0.03, 0.03)
Northeast	-0.09(-0.28, 0.09)	0.09(0.08, 0.09)
South	-0.21(-0.38, -0.03)	-0.18(-0.19, -0.16)
Southeast	-0.18(-0.36, -0.00)	-0.09(-0.09, -0.08)

This panel of figures presents the observed (black dots) and projected (fan plot) age standardized prevalence rate (per 100 000 residents) for each region in Brazil in males. The dots present the observed values between 2000-2021 and the fan plot presents the projected estimates between 2022- 2050.The line in the fan plot represents the median with the edges corresponding to the upper and lower bounds of the interquartile range. The table presents the estimated annual percentage change (with 95% confidence interval) calculated for each period. The figure for the Brazil is presented in Figure 1B in the main manuscript.

eFigure 3. Observed and projected age-standardized prevalence rate (per 100 000) for each region in females.

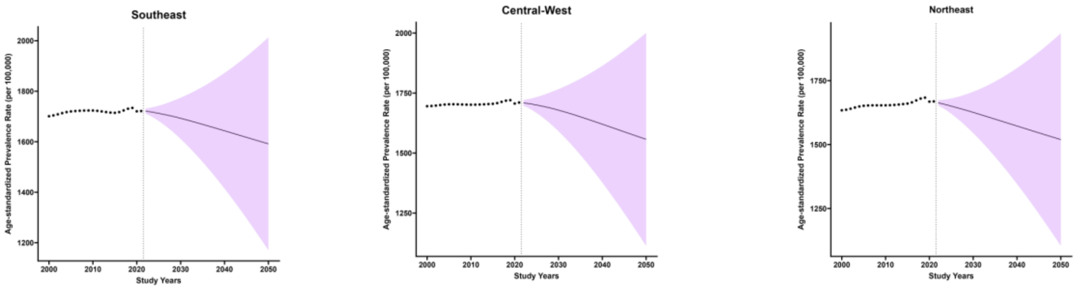

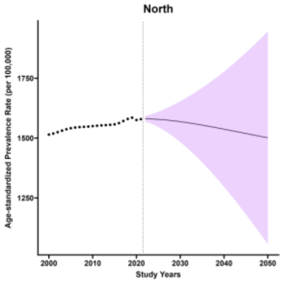

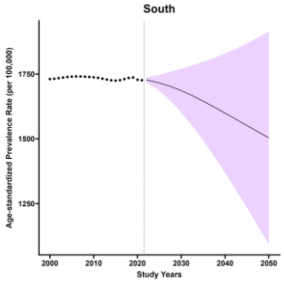

	Observed Period (2000-2021)	Projected period (2022-2050)
Region	EAPC (95% CI)	EAPC (95% CI)
Brazil	0.05(-0.11, 0.21)	-0.33(-0.34, -0.32)
Central West	0.05(-0.11, 0.21)	-0.34(-0.36, -0.33)
North	0.19(0.02, 0.35)	-0.19(-0.21, -0.18)
Northeast	0.11(-0.05, 0.27)	-0.33(-0.33, -0.32)
South	-0.02(-0.18, 0.13)	-0.51(-0.54, -0.48)
Southeast	0.04(-0.11, 0.20)	-0.29(-0.30, -0.28)

This panel of figures presents the observed (black dots) and projected (fan plot) age standardized prevalence rate (per 100 000 residents) for each region in Brazil in females. The dots present the observed values between 2000-2021 and the fan plot presents the projected estimates between 2022- 2050.The line in the fan plot represents the median with the edges corresponding to the upper and lower bounds of the interquartile range. The table presents the estimated annual percentage change (with 95% confidence interval) calculated for each period. The figure for the Brazil is presented in Figure 1C in the main manuscript.

eFigure 4. Observed and projected age-standardized disability adjusted life-years rate (per 100 000) for each region.

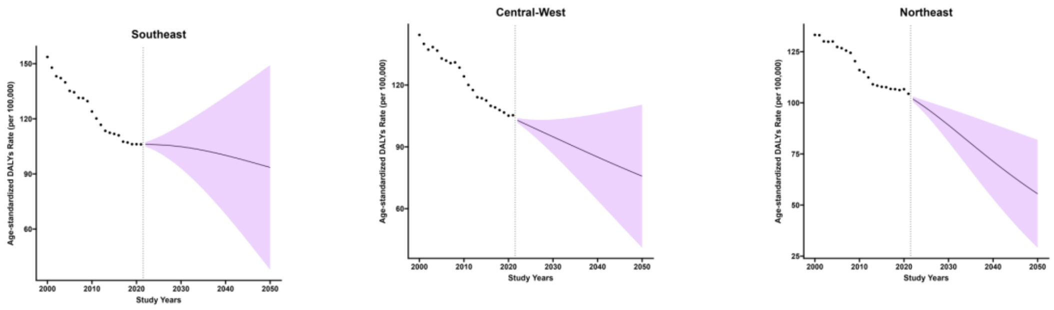

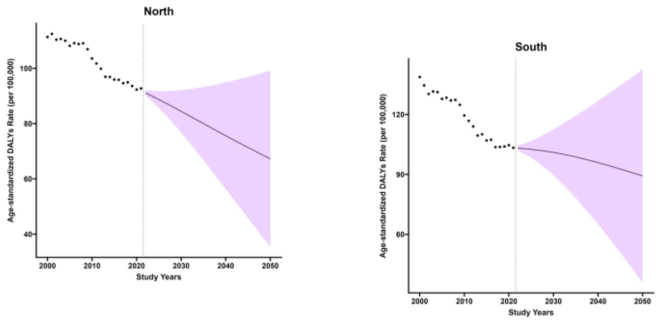

	Observed Period (2000-2021)	Projected period (2022-2050)
Region	EAPC (95% CI)	EAPC (95% CI)
Brazil	-1.58 (-2.18, -0.99)	-1.17 (-1.22, -1.11)
Central West	-1.59 (-2.18, -1.01)	-1.09 (-1.10, -1.08)
North	-1.05 (-1.69, -0.40)	-1.09 (-1.11, -1.07)
Northeast	-1.32 (-1.92, -0.72)	-2.15 (-2.25, -2.10)
South	-1.51 (-2.11, -0.91)	-0.53 (-0.57, -0.48)
Southeast	-1.85 (-2.43, -1.27)	-0.46 (-0.51, -0.41)

This panel of figures presents the observed (black dots) and projected (fan plot) age standardized disability adjusted life-years rate (per 100 000 residents) for each region in Brazil in females. The dots present the observed values between 2000-2021 and the fan plot presents the projected estimates between 2022- 2050.The line in the fan plot represents the median with the edges corresponding to the upper and lower bounds of the interquartile range. The table presents the estimated annual percentage change (with 95% confidence interval) calculated for each period. The figure for the Brazil is presented in Figure 2A in the main manuscript.

eFigure 5. Observed and projected age-standardized disability adjusted life-years rate (per 100 000) for each region in males.

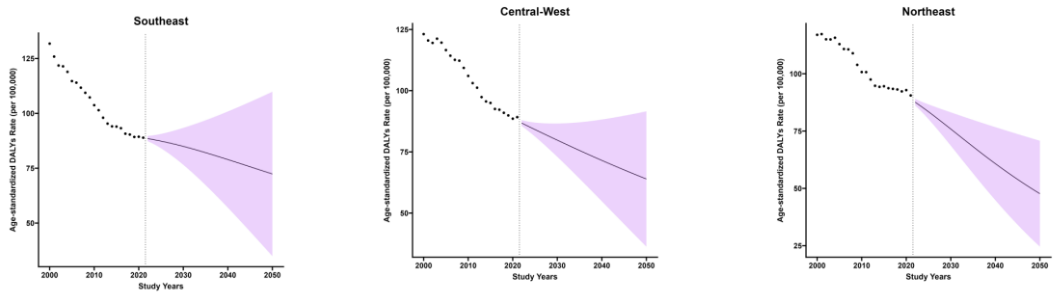

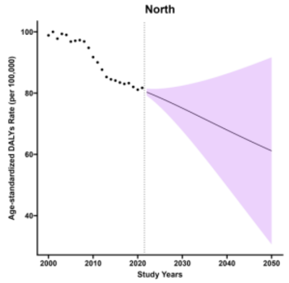

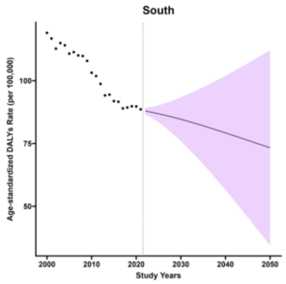

Region	Observed Period (2000-2021) EAPC (95% CI)	Projected period (2022-2050) EAPC (95% CI)
Brazil	-1.67 (-2.31, -1.02)	-1.30 (-1.34, -1.25)
Central West	-1.76 (-2.39, -1.12)	-1.09 (-1.09, -1.08)
North	-1.16 (-1.84, -0.47)	-0.98 (-1.00, -0.96)
Northeast	-1.41 (-2.05, -0.77)	-2.18 (-2.24, -2.12)
South	-1.57 (-2.21, -0.92)	-0.66 (-1.69, -0.63)
Southeast	-1.94 (-2.57, -1.30)	-0.73 (-1.76, -0.70)

This panel of figures presents the observed (black dots) and projected (fan plot) age standardized disability adjusted life-years rate (per 100 000 residents) for each region in Brazil in females. The dots present the observed values between 2000-2021 and the fan plot presents the projected estimates between 2022- 2050.The line in the fan plot represents the median with the edges corresponding to the upper and lower bounds of the interquartile range. The table presents the estimated annual percentage change (with 95% confidence interval) calculated for each period. The figure for the Brazil is presented in Figure 2B in the main manuscript.

eFigure 6. Observed and projected age-standardized disability adjusted life-years rate (per 100 000) for each region in females.

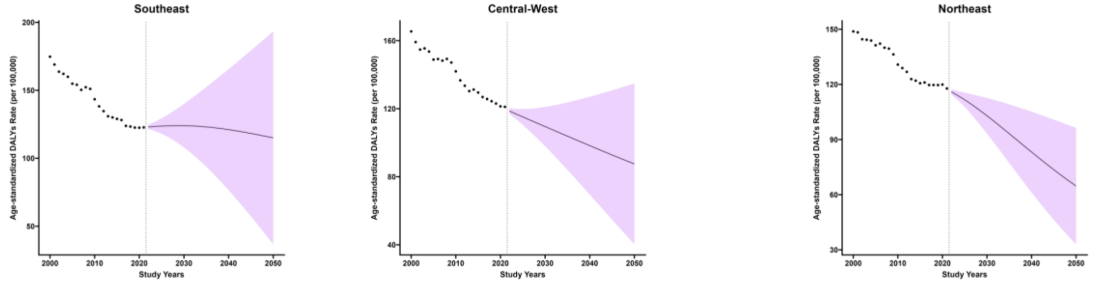

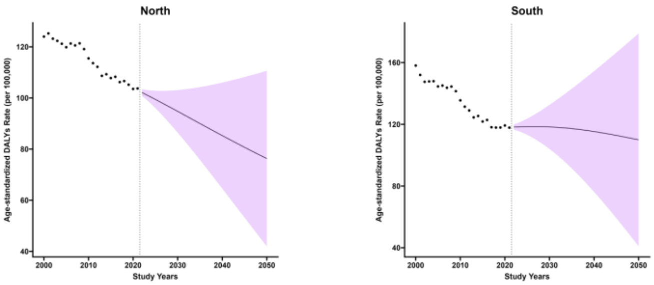

Region	Observed Period (2000-2021) EAPC (95% CI)	Projected period (2022-2050) EAPC (95% CI)
Brazil	-1.52 (-2.08, -0.96)	-1.03 (-1.11, -0.97)
Central West	-1.48 (-2.03, -0.93)	-1.08 (-1.10, -1.07)
North	-0.98 (-1.59, -0.36)	-1.04 (-1.06, -1.03)
Northeast	-1.26 (-1.83, -0.69)	-2.10 (-2.19, -2.00)
South	-1.47 (-2.03, -0.91)	-0.27 (-0.31, -0.22)
Southeast	-1.79 (-2.33, -1.24)	-0.25 (-0.31, -0.19)

This panel of figures presents the observed (black dots) and projected (fan plot) age standardized disability adjusted life-years rate (per 100 000 residents) for each region in Brazil in females. The dots present the observed values between 2000-2021 and the fan plot presents the projected estimates between 2022- 2050.The line in the fan plot represents the median with the edges corresponding to the upper and lower bounds of the interquartile range. The table presents the estimated annual percentage change (with 95% confidence interval) calculated for each period. The figure for the Brazil is presented in Figure 2C in the main manuscript.
